# Emerging recombinant noroviruses identified by clinical and waste water screening

**DOI:** 10.1038/s41426-018-0047-8

**Published:** 2018-03-29

**Authors:** Jennifer H. Lun, Joanne Hewitt, Alefiya Sitabkhan, John-Sebastian Eden, Daniel Enosi Tuipulotu, Natalie E. Netzler, Leigh Morrell, Juan Merif, Richard Jones, Bixing Huang, David Warrilow, Kelly-Anne Ressler, Mark J. Ferson, Dominic E. Dwyer, Jen Kok, William D. Rawlinson, Daniel Deere, Nicholas D. Crosbie, Peter A. White

**Affiliations:** 1 Faculty of Science, School of Biotechnology and Biomolecular Sciences, University of New South Wales, Sydney, NSW 2052 Australia; 2 Institute of Environmental Science and Research, Kenepuru Science Centre, Porirua, 5022 New Zealand; 3 Faculty of Science, School of Life and Environmental Sciences, University of Sydney, Sydney, NSW 2006 Australia; 4 Centre for Virus Research, The Westmead Institute for Medical Research, The University of Sydney, Sydney, NSW 2145 Australia; 5 SAViD (Serology and Virology Division), Department of Microbiology, Prince of Wales Hospital, Sydney, NSW 2031 Australia; 6 Douglass Hanly Moir Pathology, Macquarie Park, Sydney, NSW 2113 Australia; 7 Forensic and Scientific Services, Department of Health, Queensland Government, Archerfield, QLD 4108 Australia; 8 Public Health Unit, South Eastern Sydney Local Health District, Sydney, NSW 2217 Australia; 9 School of Public Health and Community Medicine, University of New South Wales, Sydney, NSW 2052 Australia; 10 Institute for Clinical Pathology and Medical Research, NSW Health Pathology, Westmead Hospital and University of Sydney, Sydney, NSW 2145 Australia; 11 Faculty of Medicine, School of Medical Sciences, University of New South Wales, Sydney, NSW 2052 Australia; 12 Faculty of Science, School of Biotechnology and Biomolecular Sciences, University of New South Wales, Sydney, NSW 2052 Australia; 13 Water Futures Pty Ltd, Sydney, NSW 2073 Australia; 14 Melbourne Water Corporation, Docklands, VIC 3001 Australia

## Abstract

Norovirus is estimated to cause 677 million annual cases of gastroenteritis worldwide, resulting in 210,000 deaths. As viral gastroenteritis is generally self-limiting, clinical samples for epidemiological studies only partially represent circulating noroviruses in the population and is biased towards severe symptomatic cases. As infected individuals from both symptomatic and asymptomatic cases shed viruses into the sewerage system at a high concentration, waste water samples are useful for the molecular epidemiological analysis of norovirus genotypes at a population level. Using Illumina MiSeq and Sanger sequencing, we surveyed circulating norovirus within Australia and New Zealand, from July 2014 to December 2016. Importantly, norovirus genomic diversity during 2016 was compared between clinical and waste water samples to identify potential pandemic variants, novel recombinant viruses and the timing of their emergence. Although the GII.4 Sydney 2012 variant was prominent in 2014 and 2015, its prevalence significantly decreased in both clinical and waste water samples over 2016. This was concomitant with the emergence of multiple norovirus strains, including two GII.4 Sydney 2012 recombinant viruses, GII.P4 New Orleans 2009/GII.4 Sydney 2012 and GII.P16/GII.4 Sydney 2012, along with three other emerging strains GII.17, GII.P12/GII.3 and GII.P16/GII.2. This is unusual, as a single GII.4 pandemic variant is generally responsible for 65–80% of all human norovirus infections at any one time and predominates until it is replaced by a new pandemic variant. In sumary, this study demonstrates the combined use of clinical and wastewater samples provides a more complete picture of norovirus circulating within the population.

## Introduction

Next-generation sequencing (NGS) is a new and rapidly evolving technology that facilitates the simultaneous sequencing of large amounts of genetic material. Over the past decade, NGS has advanced and revolutionised pathogen genomic sequencing, with increased turnaround times and outputs, generating more data at much lower per base costs. Owing to the availability and cost-effectiveness of NGS, it can be used as a tool to study viral genetic diversity in waste water^1^.

Molecular epidemiological studies of norovirus are usually performed with clinical samples collected from patients presenting at medical facilities. However, this is not representative of the whole norovirus population and is biased towards severe symptomatic cases, thus offering only a narrow picture of the complete viral diversity. Community norovirus infection and prolonged shedding results in high levels of norovirus in waste water^2,3^, making waste water a useful medium for population-based, epidemiological scale, enteric virus surveillance.

Norovirus is a leading cause of acute viral gastroenteritis (AGE) among people of all age groups^4^. Norovirus-associated outbreaks commonly occur in closed environments such as nursing homes, hospitals, childcare centres and cruise ships^5^. Institutional outbreaks of viral gastroenteritis are difficult to control and have significant global economic burden to public healthcare systems (USD $4.2 billion) and communities (USD $60.3 billion) each year^6^.

Norovirus is transmitted from person-to-person, mainly through the faecal-oral route. It is highly infectious^7^ and transmission is aided by its stability in the external environment. Clinical symptoms of norovirus infection include diarrhoea, vomiting, nausea, headaches, chills and abdominal cramps, usually lasting 2 to 4 days^8^. Severe and persistent symptoms can be experienced in vulnerable populations including children, immunocompromised individuals and the elderly^9^.

Norovirus is a non-enveloped, 27–35 nm virion, which contains a positive sense, single-stranded RNA genome of ~ 7500 nucleotides (nt). The *Norovirus* genus is classified within the *Caliciviridae* family and can be divided into seven genogroups (GI–GVII), and further separated into more than 40 genotypes, based on the amino acid sequence of the full-length capsid region^10^. GI, GII and GIV have been shown to infect humans; however, viruses from GII genotype 4 (GII.4) are responsible for pandemics and 65–80% of all norovirus infections globally^11,12^. Norovirus GII.4 evolution is driven through the generation of genetic variants, by antigenic drift and shift (recombination), leading to the emergence of novel and potential pandemic viruses approximately every 2–3 years^13^. Amino acid divergence in the P2 protruding domain of the capsid (VP1) contributes to the emergence of these viruses through escape from herd immunity^13,14^. Another important mechanism driving the evolution and emergence of norovirus is recombination, with the breakpoint usually found at the ORF1/ORF2 overlap region, which separates the non-structural and structural regions of the genome^15^.

Six distinct norovirus GII.4 variants have been associated with pandemics of AGE over the past two decades^15,16^. Pandemic variants are distinguished based on the complete full-length capsid amino acid sequences, where more than 5% amino acid divergence is considered indicative of a new pandemic variant. GII.4 pandemic variants are named based on the location first identified, followed by the year. The GII.4 pandemic variants include the following: US 1995/96^17^, Farmington Hills 2002^18^, Hunter virus 2004^19^, Den Haag 2006b^12^, New Orleans in 2009^20^ and, in early 2012, Sydney 2012. The GII.4 Sydney 2012 variant was first identified in March 2012 in Australia^21,22^ and originated through recombination between the GII.4 Osaka 2007 variant and GII.4 Apeldoorn 2008^13^.

Despite the predominance of norovirus GII.4, several non-GII.4 strains are also an important cause of norovirus infections. In the 2014/2015 winter in Southeast Asia, a sudden increase in gastroenteritis was observed, as the GII.17 Kawasaki strain emerged^23,24^. However, this trend was not observed in North America or Europe, with only a low reported prevalence of GII.17 infections^25,26^. In addition, norovirus GII.3 appears to have a major role in childhood infections and has been the capsid genotype most frequently associated with children for the past few decades^27^. These strains are often recombinants, in particular the GII.3 capsid is often associated with a GII.P21 ORF1 (GII.P21/GII.3)^27^, formerly termed GII.Pb/GII.3^28^.

Continuous surveillance of circulating norovirus genotypes in the community is essential to better understand the evolution and diversity of norovirus. Only through early identification of potential pandemic variants can sufficient warning be provided to public health sectors, to implement infection control measures. These include isolation of infected individuals, community health warnings and education for robust hygiene and decontamination practices. Therefore, we examined the molecular epidemiology of circulating norovirus from clinical samples collected in Australia and New Zealand (NZ) from July 2014 to December 2016, to monitor current epidemic strains, to identify emerging pandemic variants and to characterise recombinant viruses. Using NGS (Illumina MiSeq platform), the diversity of norovirus in waste water was monitored at a city-based population scale in both Sydney and Melbourne, Australia. The changing patterns of prevalent strain emergence were compared with those found in clinical samples with a particular focus on the timings of viral emergence. This study highlights the importance of multiple sample types for a more complete understanding of the norovirus diversity at a population level and establishes the relationship between clinical and waste water findings.

## Materials and methods

### Ethics statement

University of New South Wales (UNSW) Human Research Ethics Advisory Panel reviewed and approved the ethics application for this study (HC16826 and HC17459).

### Clinical specimen collection and outbreak identification

All samples were collected between July 2014 and December 2016. For Australia, clinical specimens were collected as part of routine diagnostic services requested by clinicians or public health agencies. A total of 285 norovirus positive clinical samples (New South Wales (NSW) = 270, Australian Capital Territory (ACT) = 10, Queensland (QLD) = 5) were collected from public and private laboratories. Samples were collected from sporadic gastroenteritis cases and outbreaks. For NZ, clinical samples were collected as part of the NZ Ministry of Health norovirus outbreak surveillance programme. Samples were referred to the Norovirus Reference Laboratory at the Institute of Environmental Science and Research by diagnostic community or hospital laboratory, or directly through public health agencies. At least one sample from each gastroenteritis outbreak that was positive for norovirus (*n* = 497) was genotyped. An outbreak is defined when two or more cases are linked by time, location or food sources, and confirmed to be the same genotype. All other samples are categorised as sporadic gastroenteritis cases.

### Waste water sample collection

Influents of Bondi and Malabar waste water treatment plants (WWTPs) from Sydney, Australia (250 mL), and Western Treatment Plants in Werribee, Melbourne (1 L) (population capacities of 296,350, 1,667,460 and 2,400,000, respectively), were collected each month from January to December 2016. All samples were delivered to UNSW on the day of collection and stored at – 80 °C upon arrival.

### **Viral concentration and RNA extraction**

For clinical samples collected in Australia, 20% (v/v) suspensions in water were prepared. Viral RNA extraction was performed using QIAamp Viral RNA mini kit (Qiagen, Hilden, Germany) following manufacturer’s instructions^12^. For clinical samples collected in NZ, 20% (v/v) suspensions were prepared and clarified with chloroform as previously described^29^. Viral RNA extraction was performed using the Roche Viral Nucleic Acid Extraction Kit (Roche, Basel, Switzerland) in accordance to the manufacturer’s instructions.

Waste water samples (12 mL) were centrifuged at 9400 × *g* at room temperature for 15 min to remove debris. The supernatant was ultra-centrifuged at 186,000 × *g* at 4 °C for 1.5 h to concentrate virus and the pellet resuspended in 100 µL of phosphate-buffered saline. Viral RNA extraction was performed using QIAamp Viral RNA mini kit (Qiagen)^12^.

### **MS2 process control**

MS2 bacteriophage was used as extraction and process control for Australian clinical and waste water samples. MS2 is a useful control when spiked into clinical and environmental samples to validate all downstream processes including viral RNA nucleic acid extraction, reverse transcription and PCR amplification^30^. MS2 was prepared as 20 µL frozen aliquots with a concentration of 2.57 × 10^5^± 1.61 × 10^5^ genome copies of viruses, which produced a cycle threshold (ct) value of 16.26 ± 1.6 when spiked into clinical and waste water samples (*n* = 40).

### RT-PCR of norovirus RNA from clinical and waste water samples

For Australian clinical samples, norovirus detection was first performed using reverse-transcription PCR (RT-PCR) targeting the 5′-end of capsid gene (region C) of norovirus GI and GII, as described previously^13^. Sequence from the 5′-end of norovirus capsid gene allows differentiation of genogroups and genotypes, and the primers used have previously been shown to be able to amplify and distinguish a wide range of genotypes^31,32^. For the detection of potential recombinant norovirus GII strains in Australia, a 575 bp region spanning the ORF1/ORF2 overlap was amplified^33^. Full-length norovirus genomes from three samples collected from NSW were amplified as described in Eden et al.^21^. For NZ samples, a norovirus GI and GII duplex RT-quantitative PCR followed by either a norovirus GI or GII RT-PCR that spanned the norovirus region B (within ORF1) and region C, across the ORF1/2 overlap, was used for genotyping as described previously^29^. PCR products were purified and sequenced as previously described^29^.

To assess antigenic variation within the capsids of circulating strains, the full-length capsid gene sequences of representative GII.2, GII.3 and GII.4 strains were amplified^21^.

In preparation for MiSeq sequencing of waste water amplicons, a second-round PCR was performed to attach Illumina sequencing adapters; this was carried out in accordance to the manufacturer’s protocol. With the addition of Illumina overhang adapters, the final amplicon size is 373 bp. All PCR amplicons were cleaned up using Agencourt AMPure XP beads (Beckman Coulter, California, USA).

### Full-length norovirus genome sequencing preparation

For the norovirus full-length genome sequencing of six QLD samples, a second method was used. RNA was extracted from a 10% (v/v) faecal suspension using QIAamp viral RNA extraction kit (Qiagen). DNase Heat&Run (ArcticZymes, Tromsø, Norway) was used to remove host and other contaminating DNA. First-strand cDNA was prepared using Protoscript II kit (New England Biolabs, Massachusetts, USA) followed by second-strand DNA synthesis using a cocktail of *Escherichia coli* DNA ligase, DNA polymerase I and RNase H (NEB).

### Nextera XT library preparation for NGS

Illumina libraries were prepared using the Nextera XT DNA sample preparation kit and quantified using the Quant-iT PicoGreen dsDNA assay kit. The fragment sizes were evaluated using Tape Station D1000 (Agilent Technologies, California, USA). Libraries generated from waste water and three NSW strains were submitted to Ramaciotti Centre for Genomics (UNSW) for paired-end sequencing on the Illumina MiSeq platform using a v2 300 cycle kit (2 × 150 bp). For the six QLD strains, libraries were sequenced using the v2 mid-output kit on a NextSeq 500 machine in the Public Health Virology Laboratory, QLD.

### **Norovirus phylogenetic analysis**

Phylogenetic analyses of the partial polymerase (172 bp) and capsid regions (282 bp) were used to determine norovirus GII genotypes identified in clinical samples. For GI genotypes, 172 bp and 223 bp of partial polymerase and capsid regions were used for phylogenetic analysis. Polymerase and capsid sequences were aligned separately using MUSCLE and compared with known reference sequences using maximum likelihood phylogenetic analysis^34^ and confirmed with an online genotyping tool (NoroNet)^35^.

### NGS data analysis

Sequences were removed if they did not range between 200 and 320 bp. The paired-end sequences were merged and aligned to norovirus reference sequences. A minimum of two representative reference sequences of each norovirus genotype were used. The parameters were as default for the Geneious mapper, v 9.0.5 (Geneious software R9, Biomatters, Auckland, New Zealand) and medium sensitivity was used for reference mapping and virus identification. The number of reads aligned to each reference genotype was used to quantify the abundance of each respective genotype within waste water samples. The proportion representation of each genotype was then calculated by dividing the number of reads attributed to the genotype over the total number of sequencing reads.

### Identification of recombination breakpoints

Assembly of the reads generated from nine norovirus full-length genome sequencing was performed using Geneious software R9, by mapping reads to norovirus reference sequences. The full-length genome consensus sequences were then analysed using SimPlot (v3.5) to identify recombination breakpoints^36^.

## Results

**Fig. 1:**
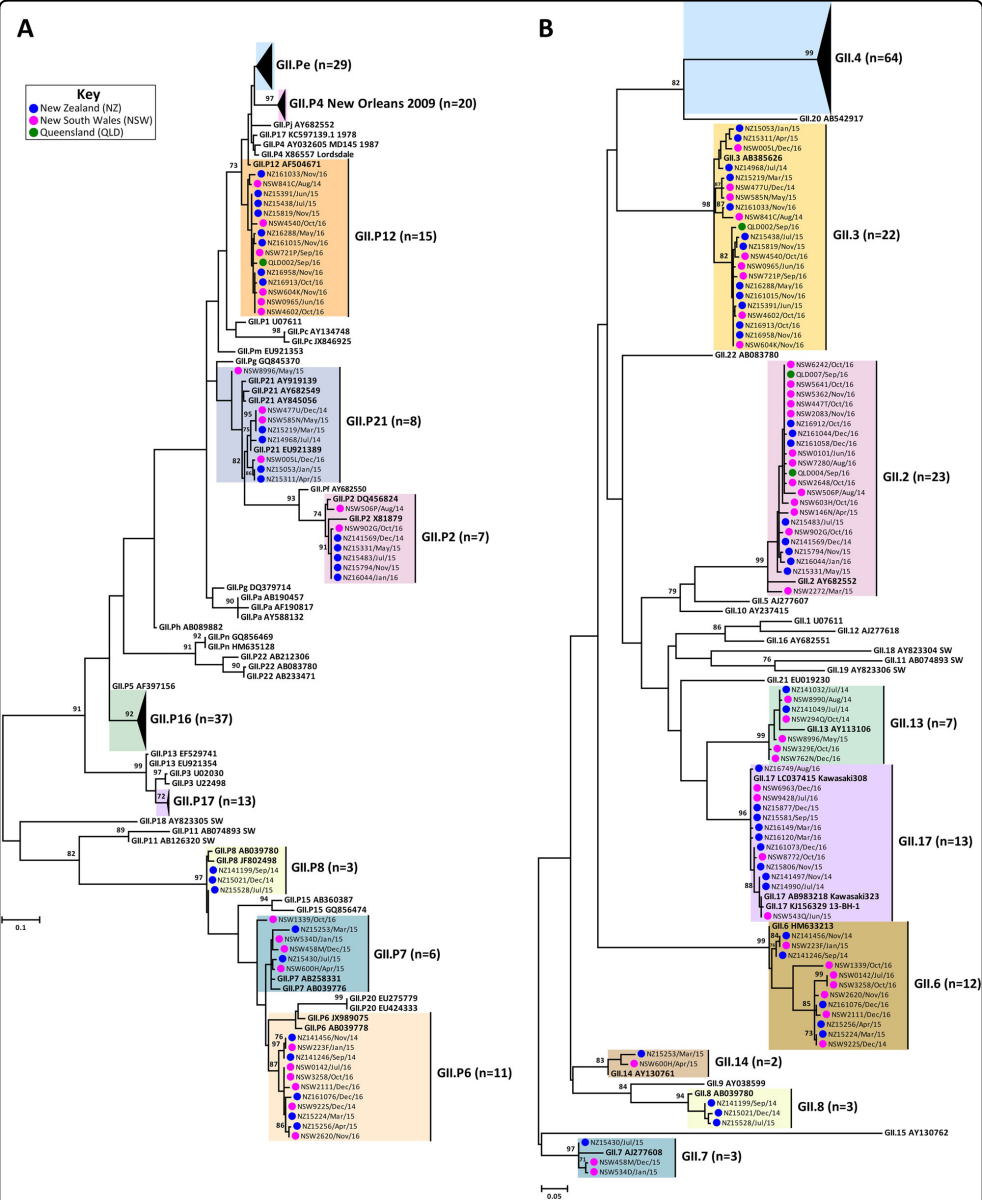
**Phylogenetic analysis of ORF1/ORF2 overlap region of GII norovirus.** Representative norovirus GII strains isolated in clinical samples (*n* = 149/686) were analysed phylogenetically. Strains analysed are denoted with a bullet (•) and colour-coded to show sample origin (pink = NSW, blue = NZ and green = QLD). Sample names contain the geographical location and time of collection. Reference strains were downloaded from GenBank and labelled with their genotype and accession number. **a** Phylogenetic analysis of 172 bp of the 3′-end of the polymerase gene of norovirus GII viruses. **b** Phylogenetic analysis of 282 bp from the 5′-end of the capsid gene of norovirus GII viruses. The scale bar indicates the number of nucleotide substitutions per site. Sequence alignments were performed using the MUSCLE algorithm. Maximum likelihood phylogenetic trees were produced with MEGA 7 software^59^ and bootstrap tests (1000 replicates) based on the Kimura two-parameter model^60^. The bootstrap percentage values are shown at each branch point for values ≥ 70%

**Fig. 2:**
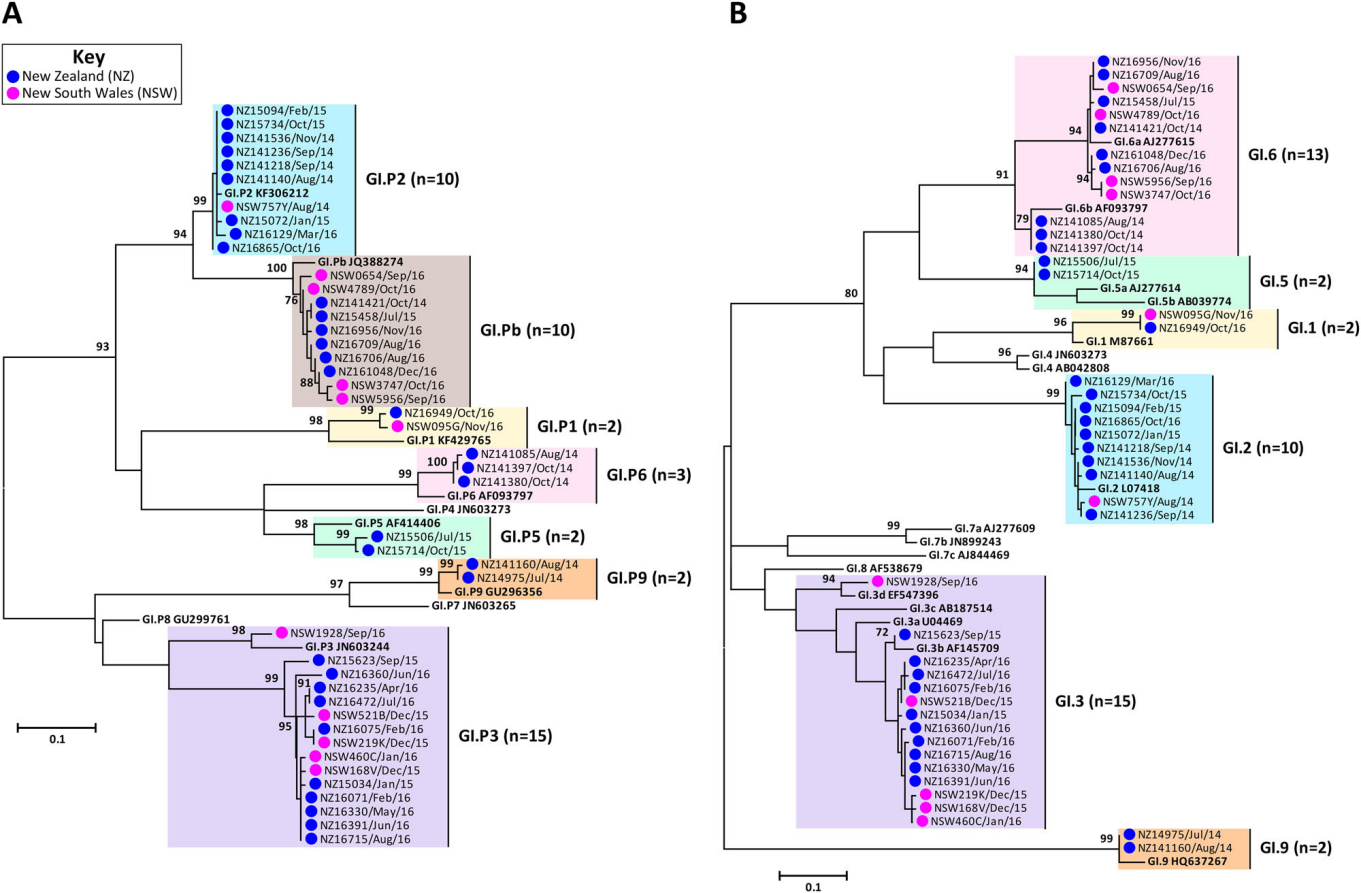
**Phylogenetic analysis of ORF1/ORF2 overlap region of GI norovirus.** Representative norovirus GI strains isolated in clinical samples (*n* = 52/83) are shown in this phylogenetic analysis. Strains analysed in this study are denoted with a bullet (•) and colour-coded to show sample origin (pink = NSW and blue = NZ). All sample names contain the geographical location and time of collection. Reference strains were downloaded from GenBank and labelled with their genotype and accession number. **a **Phylogenetic analysis of 172 bp of the 3′-end of the polymerase gene of norovirus GI viruses. **b** Phylogenetic analysis of 223 bp of the 5′-end of the capsid gene of norovirus GI viruses. The scale bar indicates the number of nucleotide substitutions per site. Sequence alignments were performed using the MUSCLE algorithm. Maximum likelihood phylogenetic trees were produced with MEGA 7 software^59^ and bootstrap tests (1000 replicates) based on the Kimura two-parameter model^60^. The bootstrap percentage values are shown at each branch point for values ≥ 70%

The prevalence of circulating norovirus GI and GII genotypes in Australia and NZ were determined in this study, which included a total of 782 norovirus-positive specimens. Of these, 285 norovirus-positive samples were collected from Australia (NSW, ACT and QLD), of which 238 were associated with sporadic cases and 47 were linked to outbreaks. In NZ, 497 laboratory-confirmed norovirus outbreaks, where representative samples were successfully genotyped, were included in this study (Table 1). Polymerase and capsid genotypes were determined by phylogenetic analysis of sequences using the maximum-likelihood method^34^ and confirmed using database searches (Table 1). To compare the identified norovirus sequences, a subset of 89 Australian (GI = 11 and GII = 78) and 104 NZ (GI = 33 and GII = 71) representative sequences were selected for phylogenetic tree construction (Figs. 1 and 2). All norovirus sequences were submitted to GenBank database, accession numbers MG585752-MG585937.

**Table 1:** Total number of norovirus genotypes identified in Australia and NZ from July 2014 to December 2016

Genotype		No. of outbreak cases		No. of sporadic cases
Polymerase	Capsid	NZ (%)	AUS^1^ (%)	AUS^2^ (%)
Undetermined	GI.3	–	–	2 (0.8)
Undetermined	GI.6	–	1 (2.1)	3 (1.3)
Undetermined	GI.9	1 (0.2)	–	–
GI.P1	GI.1	2 (0.4)	–	1 (0.4)
GI.P2	GI.2	21 (4.2)	–	1 (0.4)
GI.P3	GI.3	22 (4.4)	–	5 (2.1)
GI.P5	GI.5	2 (0.4)	–	–
GI.Pb	GI.6	8 (1.6)	–	–
GI.P6	GI.6	7 (1.4)	3 (6.4)	–
GI.P7	GI.7	1 (0.2)	–	–
GI.P9	GI.9	3 (0.6)	–	–
Undetermined	GII.2	–	–	2 (0.8)
GII.Pe	GII.2	–	–	1 (0.4)
GII.P2	GII.2	17 (3.4)	–	2 (0.8)
GII.P16	GII.2	27 (5.4)	4 (8.5)	41 (17.2)
GII.P21	GII.3	11 (2.2)	1 (2.1)	4 (1.7)
GII.P12	GII.3	37 (7.4)	2 (4.3)	21 (8.8)
Undetermined	GII.3	2 (0.4)	–	–
Undetermined	GII.4 Sydney 2012	6 (1.2)	–	–
GII.P16	GII.4 Sydney 2012	20 (4.0)	12 (25.5)	25 (10.5)
GII.Pe	GII.4 Sydney 2012	180 (36.2)	18 (38.3)	60 (25.2)
GII.P4 NO 2009	GII.4 Sydney 2012	39 (7.8)	4 (8.5)	38 (16.0)
Undetermined	GII.6	2 (0.4)	–	–
GII.P7	GII.6	31 (6.2)	1 (2.1)	15 (6.3)
Undetermined	GII.7	5 (1.0)	–	–
GII.P7	GII.7	5 (1.0)	–	2 (0.8)
GII.P8	GII.8	2 (0.4)	–	–
GII.P3	GII.13	1 (0.2)	–	–
GII.P16	GII.13	2 (0.4)	–	4 (1.7)
GII.P21	GII.13	–	–	1 (0.4)
GII.P7	GII.14	1 (0.2)	–	1 (0.4)
GII.P17	GII.17	27 (5.4)	1 (2.1)	9 (3.8)
ND	GII.20	1 (0.2)	–	–
GII.P7	ND	1 (0.2)	–	–
Mixed infection	Mixed infection	8 (1.6)	–	–
Undetermined	Undetermined	5 (1.0)	–	–
Total		497	47	238

^1^AUS outbreak samples include NSW (*n *= 37), ACT (*n *= 7) and QLD (*n *= 3)

^2^AUS acute gastroenteritis samples include NSW (*n *= 233), ACT (*n *= 3) and QLD (*n *= 2)

*ACT* Australian Capital Territory, *AUS* Australia, *NSW* New South Wales, *QLD* Queensland, *NZ* New Zealand

### Norovirus GII.4 distribution in Australia and New Zealand

**Fig. 3:**
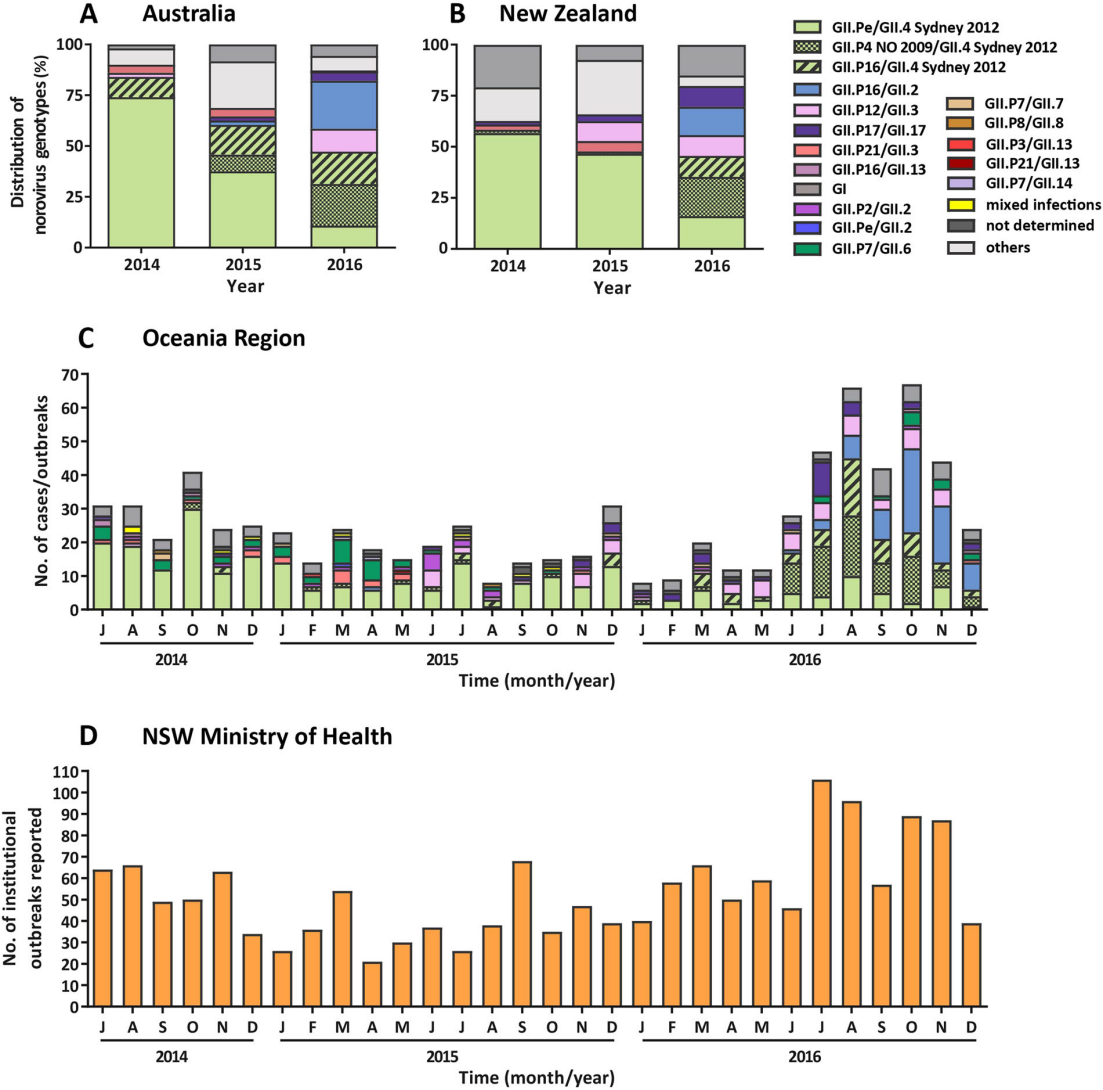
**Yearly and monthly distributions of norovirus genotypes identified in the Oceania region, compared with institutional outbreaks reported to NSW Ministry of Health between July 2014 to December 2016.** **a** The prevalence of norovirus genotypes identified in Australia during the study period. A total of 238 individual norovirus cases and 47 outbreaks were sequenced and genotyped from Australia (NSW, ACT and QLD). **b** All samples collected, sequenced and genotyped from New Zealand represented viruses from 497 separate outbreaks. Samples with unknown capsid genotypes and mixed GI/GII infections were excluded in this analysis (*n* = 14). **c** The monthly norovirus genotypic distribution for Australia and New Zealand combined throughout the study period (2014–2016) was examined. Different genotypes and GII.4 variants are colour-coded as per the legends. All norovirus GI viruses identified are grouped together under GI (dark grey) and others include all GII viruses identified (GII.P2/GII.2, GII.Pe/GII.2, GII.P7/GII.6, GII.P7/GII.7, GII.P16/GII.13 and GII.P21/GII.13) that are not indicated in the legends. **d** The monthly number of institutional outbreaks reported to the NSW Ministry of Health, Australia

A decline in the prevalence of GII.Pe/GII.4 Sydney 2012 was observed over the study period in both Australia and NZ (Fig. 3). Specifically, in Australia in 2014 (July to December), the Sydney 2012 variant represented 74.0% of all noroviruses, which decreased to 37.5% in 2015 and only accounted for 10.8% of noroviruses in 2016 (Table 1 and Fig. 3a). This was also observed in NZ, with the Sydney 2012 variant responsible for 56.7% of norovirus outbreaks from July to December 2014, 46.6% in 2015 and 16.0% in 2016 (Table 1 and Fig. 3b).

Although the GII.Pe/GII.4 Sydney 2012 variant itself exhibited a rapid decline over the study’s time frame, viruses with a Sydney GII.4 capsid remained prevalent throughout 2016, representing 47.2% and 45.5% of the total norovirus population in Australia and NZ, respectively (Fig. 3c). This was resultant of the two newly emerged GII.4 Sydney-derived recombinant forms. The first recombinant, GII.P16/GII.4 Sydney 2012, was initially identified in 2015 in Australia and accounted for 14.6% and 15.9% of norovirus identified in 2015 and 2016, respectively. It was the third most predominant norovirus in circulation in 2016 (Fig. 3a). Although this recombinant first emerged in August 2015 in NZ, the next outbreak associated with GII.P16/GII.4 Sydney 2012 was not identified until April 2016 and accounted for only 10.2% of outbreaks in 2016 (Fig. 3b). The second Sydney recombinant detected, GII.P4 New Orleans 2009/GII.4 Sydney 2012 (GII.P4 NO 2009/GII.4 Sydney 2012), emerged in Australia in May 2015 and represented 8.3% of cases, which increased to 20.5% in 2016 (Fig. 3a).

Similarly, in NZ, the GII.4 NO 2009/Sydney 2012 strain, emerged in February 2013 (with two outbreaks)^33^, was the predominant (36/187, 19.3%) norovirus detected in outbreaks in 2016. Of these 35 outbreaks, 23 were identified in nursing homes or hospital wards between July and September 2016. The second most frequently detected norovirus was the GII.Pe/GII.4 Sydney 2012 variant (30/187, 16.0%) (Fig. 3b). The emergence of these new viruses was accompanied by an increase in institutional outbreaks of gastroenteritis reported to the NSW Ministry of Health, particularly during the winter period (June–August) of 2016 (Fig. 3d).

### Other emerging noroviruses

The GII.17 Kawasaki strain was first identified in June 2015 in Australia (Table 2) and accounted for 2.1% of 2015 norovirus cases and 4.6% in 2016, including one outbreak (Table 1 and Fig. 3a). In NZ, the GII.17 strain was identified in April 2014 and was responsible for 1.7% (*n* = 3) of all outbreaks that year, followed by 3.4% in 2015 and 10.2% in 2016 (Table 1 and Fig. 3b).

**Table 2:** Full-length genome sequences of novel noroviruses viruses identified in this study and their reported outbreak dates

Genotype (polymerase/capsid)	Sample ID of full-length sequences	GenBank accession no.	Date of collection	Sequence length (bp)	Australia			New Zealand	
					First case	First outbreak	Outbreak setting	First outbreak	Outbreak setting
GII.P17/GII.17	NSW9428	KY905330	Jul 2016	7491	Jun 2015	Jul 2016	Residential	Jul 2014	Nursing home
GII.P17/GII.17	NSW543Q	KY905332	Jun 2015	7533					
GII.Pe/GII.4 Sydney 2012	QLDB101	KY905333	Sep 2016	7497	Mar 2012	Apr 2012	Hospital	Feb 2012	Nursing home
GII.P4 NO 2009/GII.4 Sydney 2012^1^	NSW789Z	KY905331	Aug 2016	7546	May 2015	Aug 2016	Cruise ship	Oct 2014	Nursing home
GII.P16/GII.4 Sydney 2012^1^	QLDB309	KY905335	Sep 2016	7529	Jul 2015	Jul 2016	Cruise ship	Aug 2015	Nursing home
GII.P12/GII.3^1^	QLDB207	KY905334	Sep 2016	7498	Aug 2014	Jun 2016	Residential	Apr 2015	Commercial food operator
GII.P16/GII.2^1^	QLDB411	KY905336	Sep 2016	7527	Apr 2015	Aug 2016	Residential	Mar 2016	School/college
GII.P16/GII.2	QLDB512	KY905337	Sep 2016	7522					
GII.P16/GII.2	QLDB614	KY905338	Oct 2016	7522					

^1^ Sequences selected for Simplot analysis in Fig. 5

In addition to the GII.4 Sydney recombinants, a novel GII.P16/GII.2 recombinant emerged in Australia in April 2015 (Table 2). Its prevalence steadily increased in 2016, accounting for 23.6% of all noroviruses detected that year, including three outbreaks (Table 1 and Fig. 3a). In NZ, this recombinant was first identified from an outbreak in March 2016, and that year the strain accounted for 13.9% of all norovirus outbreaks (Table 1 and Fig. 3b).

The GII.P21/GII.3 recombinant, often associated with childhood infections, was seen in Australia in both 2014 (4.0%) and 2015 (4.2%); however, it only accounted for one case in 2016 (Fig. 3a). This trend was also observed in NZ, where it accounted for 7.6% of all outbreaks in 2014/15 (of which 63.6% were associated with childcare facilities) but had disappeared by June 2015 (Fig. 3b). In Australia, concomitant with the GII.P21/GII.3 decline, a second emerging virus (GII.P12/GII.3) was identified in 2016 (11.3% of cases) and was largely associated with childhood infections (20/22 cases). This virus was identified in NZ in 2015 (9.7% of outbreaks) and 2016 (10.2% of outbreaks). In contrast, only one-third of those outbreaks occurred in childcare centres, with the majority in long-term care facilities (48.6%).

### Genogroup I norovirus

In Australia, GI accounted for only 5.5% of all norovirus cases, with four different GI capsid genotypes identified. Norovirus GI.3 (*n* = 7/16) and GI.6 (*n* = 7/16) were the most common, followed by GI.2 (*n* = 1/16) and GI.1 (*n* = 1/16) (Table 1 and Fig. 1). In comparison, norovirus GI was more prevalent in NZ and accounted for 13.3% of the 497 norovirus-associated outbreaks. A total of six wild-type GI strains were identified; GI.P3/GI.3 as the most prevalent (22/66, 33.3%), followed by GI.P2/GI.2 (21/66, 31.8%) and GI.P6/GI.6 (7/66, 10.6%) (Table 1). A recombinant strain, GI.Pb/GI.6, was also identified in four outbreaks (Table 1).

### Outbreak settings in Australia and New Zealand

A total of 544 norovirus-associated AGE outbreaks were identified during the study period. In Australia, norovirus GI (all GI.6) and GII were responsible for 4 and 43 outbreaks, respectively (NSW = 37 outbreaks, ACT = 7 and QLD = 3) (Table 1 and Fig. 3a), whereas in NZ norovirus GI and GII were responsible for 66 and 426 outbreaks, respectively, with the genogroup of 5 outbreaks not determined (Table 1 and Fig. 3b). In NZ, GI outbreaks were caused by GI.P3/GI.3 (33.3%), followed by GI.P2/GI.2 (31.8%) and GI.Pb/GI.6 (12.1%).

The pandemic Sydney 2012 variant was the most commonly detected outbreak virus in both Australia (18/47, 38.3%) and NZ (180/497, 36.2%), across the 2.5-year study period. In Australia, the second most prevalent outbreak strain was the new GII.P16/GII.4 Sydney 2012 recombinant (23.4%), whereas GII.P4 NO 2009/GII.4 Sydney 2012 was the second most common outbreak virus in NZ (7.8%) (Table 1).

**Fig. 4:**
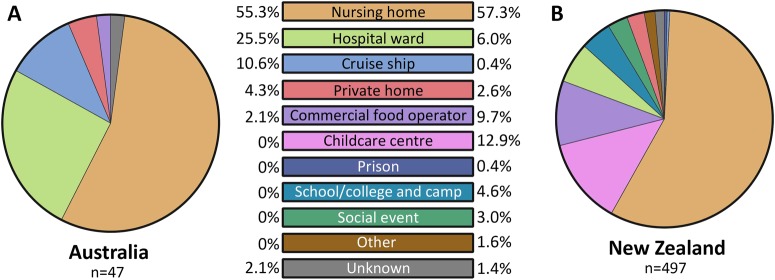
**Norovirus outbreak settings identified in Australia and New Zealand between July 2014 and December 2016.** The setting for norovirus outbreaks was compared between Australia and New Zealand during the study period. A total of 47 and 497 outbreaks were identified in Australia and New Zealand, respectively. An outbreak is defined as two or more cases linked by location and time

The majority of outbreaks were reported in nursing homes in both Australia (55.3%) and NZ (57.3%) (Fig. 4). In Australia, hospital wards were the second most common setting for reported norovirus outbreaks (12/47, 25.5%), followed by cruise ships (5/47, 10.6%) and private homes (2/47, 4.3%) (Fig. 4a). In NZ, a wider range of outbreak settings were identified, with childcare centres as the second most common setting (64/497, 12.9%), followed by commercial food operators (48/497, 9.7%) (Fig. 4b). Eight (1.6%) outbreaks were identified as mixed GI and GII genotypes infection, all detected in NZ.

### Recombination breakpoint identification in norovirus full-length genomes

**Fig. 5:**
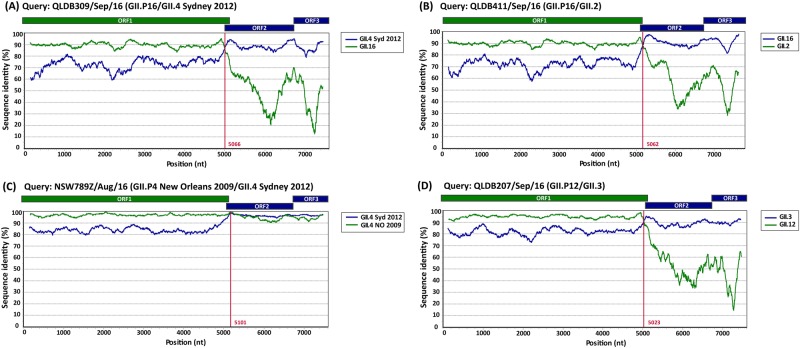
**Simplot analysis of novel norovirus recombinant strains.** Representative sequences of emerging viruses were subjected to full-length genome sequencing and analysed using Simplot for the identification of recombination breakpoints. For all recombinant strains, a single breakpoint was identified at the ORF1/ORF2 overlap region. **a** Simplot for recombinant strain GII.P16/GII.4 Sydney 2012 (QLDB309/Sep/16, GenBank accession number KY905335) with a breakpoint identified at nucleotide position 5066. **b** Simplot for recombinant strain GII.P16/GII.2 (QLD411/Sep/16, GenBank accession number KY905336) with a breakpoint identified at nucleotide position 5062. **c** Simplot for recombinant GII.P4 New Orleans 2009/GII.4 Sydney 2012 (NSW789Z/Aug/16 (GenBank accession number KY905331) with a single breakpoint identified at nucleotide position 5101. **d** Simplot for recombinant GII.P12/GII.3 (QLDB207/Sep/16, GenBank accession number KY905334) with a breakpoint at nucleotide position was 5023. The breakpoint positions are shown by red lines. Each analysis used a window size of 300 nt and a step size of 5 nt. The reference strains used are as follows: GII.4 Syd 2012 for NSW0514/2012/AU (GenBank accession number JX459908), GII.4 NO 2009 for NSW001P/2008/AU (GenBank accession number GQ845367), GII.3 for HK71/1978/CHN (GenBank accession number JX846924), GII.12 for 04-179/2005/JP (GenBank accession number AB220922), GII.16 for Neustrelitz260/2000/DE (GenBank accession number AY772730) and GII.2 for KL109/1978/MYS (GenBank accession number JX846925)

To further characterise clinically important strains, nine norovirus full-length genomes of representative viruses were analysed using Simplot to identify recombination breakpoints (Fig. 5). The consensus sequences of norovirus-assembled reads were used for each virus with coverage ranging between 8.2 × 10^6^ and 10.4 × 10^6^. This analysis revealed that both GII.17 viruses were shown to be wild-type viruses with between 99.5% and 99.7% identity to the prototype GII.17 Kawasaki-323 sequence. The other five viruses analysed were either intra-genotypic (GII.Pe/GII.4 Sydney 2012 and GII.P4 NO/GII.4 Sydney 2012) or inter-genotypic (GII.P16/GII.4 Sydney 2012, GII.P16/GII.2 and GII.P12/GII.3) recombinants with breakpoints identified at the ORF1/ORF2 overlap between nucleotide positions 5023 and 5101 (Fig. 5).

### **Norovirus capsid genotype diversity in Australian waste water samples**

**Fig. 6:**
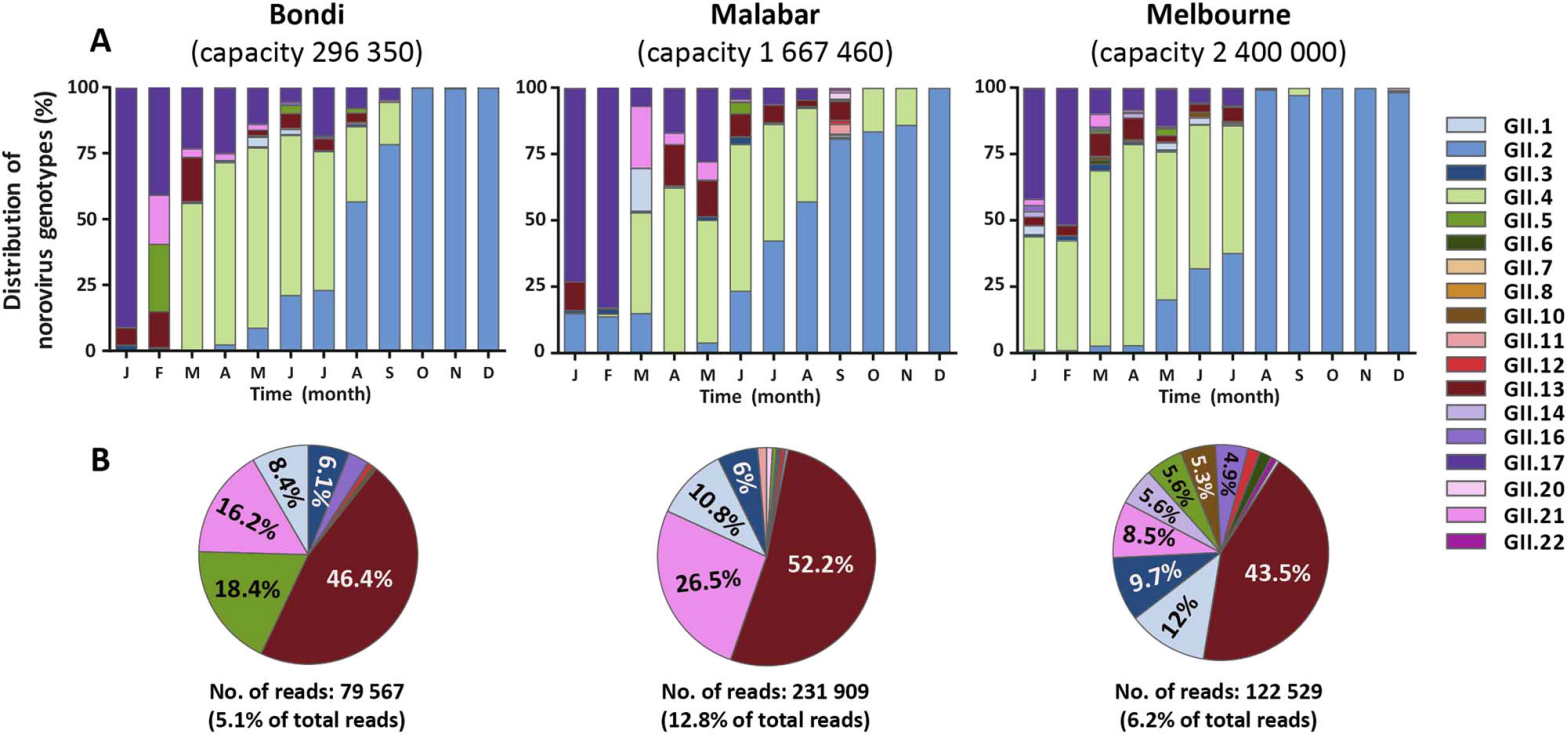
**Norovirus genotype distribution in wastewater samples collected from Sydney and Melbourne, 2016.** Norovirus genotypic distribution was determined in waste water samples by capsid amplicon sequencing using next-generation sequencing (NGS) technology. Samples were sequenced on the MiSeq platform and Geneious was used for merging and mapping of the reads to the reference sequences. Capsid genotypes are labelled in different colours as indicated by the legends. **a** The genotype distribution of norovirus capsids identified in two waste water treatment plants in Sydney (Bondi and Malabar) and one in Melbourne, Australia. The Y-axis represents the percentage norovirus distribution in each sample and the time is indicated on the X-axis. **b** To further investigate the less predominant capsid genotypes, the three most predominant capsid genotypes (GII.2, GII.4 and GII.17) across all sites were removed and the distribution of the remaining genotypes plotted as pie charts. The number of reads attributed to non-GII.2, GII.4 and GII.17 are listed below the pie chart

This study aimed to capture a complete picture of norovirus diversity at a population level, with the use of NGS technologies on waste water samples from two major Australian cities, Sydney and Melbourne. Norovirus PCR amplicons (306 bp) were generated monthly for 2016 from Malabar, Melbourne and Bondi waste water samples, and were subjected to Illumina MiSeq sequencing. An average of 588,775 reads (range 105,082–1,452,622) were generated from each collection point. All raw data were submitted to Sequence Read Archive (SRA) database, accession no. PRJNA417367. Reads were merged and assembled to known reference strains using Geneious and their relative abundance determined based on mapped read counts. Eighteen norovirus capsid genotypes were identified across all three sites and the dominant variants included were GII.4, GII.17, GII.2, GII.13, GII.3 and GII.1 (Fig. 6).

Interestingly, a decline in the proportion of GII.17 as a percentage of total reads was observed from early 2016. From its peak of 68.6% of the total WWTP norovirus population in January (average of all three sites), it decreased to 16.6% by April and accounted for only 4.1% in August 2016 (Fig. 6). This reduction was accompanied by an overall increase in the GII.4 population from March (53.4%), which was steady until the emergence of the GII.2 norovirus strain (Fig. 6). An increase of GII.2 was observed in all three sites from May 2016 onwards. In Malabar, GII.2 steadily increased from May (4.0%), July (42.5%) and September (80.8%) to December (99.9%), replacing GII.4 as the predominant strain identified (Fig. 6a). Similarly, GII.2 strains accounted for < 3% in the first months of 2016 in Melbourne, increased to 32.1% in June and to 98.3% in December. This GII.2 increase was also observed in Bondi: 2.5% in April, 56.7% in August and 99.7% in November (Fig. 6a).

## Discussion

In order to design effective control strategies such as vaccines, it is important to understand the prevalence and diversity of current and emerging noroviruses. The genotypes of circulating norovirus strains were determined in Australia and NZ between July 2014 and December 2016 in clinical samples. However, as people infected with norovirus would not necessarily seek medical advice, this can create a bias towards severe symptomatic cases and therefore does not fully represent circulating noroviruses within a population, with less pathogenic strains perhaps under-represented. The application of NGS to monitor norovirus population diversity in waste water can provide a better understanding of norovirus epidemiological data at the population scale by screening waste water produced from many thousands or millions of people. Moreover, the comparison of clinical and waste water data allows a more complete representation of norovirus epidemiology at a city-based population level.

Sequencing and phylogenetic analysis of the polymerase and capsid overlapping regions revealed the Sydney pandemic variant (GII.Pe/GII.4 Sydney 2012) remained the predominant strain in the Oceania region in 2014 (65.4% of norovirus outbreaks and cases) and 2015 (42.1%). The Sydney 2012 variant emerged in 2012 and over the ensuing 4 years herd immunity in the population is likely to have accumulated against the virus. This could account for the sudden decline of GII.4 Sydney pandemic variant in 2016 (only 13.4% of norovirus cases), concomitant with the emergence of five new viruses including two novel GII.4 Sydney 2012 recombinants, GII.P12/GII.3, GII.P16/GII.2 and GII.17. The emergence of new viruses is likely to be responsible for the increase of institutional gastroenteritis outbreaks reported to the NSW Ministry of Health between July and November 2016.

The maintenance of the GII.4 Sydney 2012 capsid in the population was facilitated by two separate recombination events, resulting in the emergence of the intra-genotypic GII.P4 NO 2009/GII.4 Sydney 2012 and the inter-genotypic GII.16/GII.4 Sydney 2012 recombinant viruses. These two recombination events likely provided the viruses a selective advantage to evade host immunity directed towards the GII.Pe-encoded non-structural region of the Sydney variant (GII.Pe/GII.4 Sydney 2012). The GII.P4 NO 2009/GII.4 Sydney 2012 virus arose, although an unusual recombination event between a pandemic variant and its pandemic predecessor^37^. In Europe, this strain was identified in late 2012 in the UK and Denmark^38,39^. However, this strain was first identified in early 2013 in NZ (data not shown) and May 2015 in Australia, but remained at low prevalence throughout 2015. It was not until 2016 when it became a major cause (19.6%) of gastroenteritis within the Oceania region. This timing is consistent with a study by Bruggink et al^40^. in Victoria, Australia, who showed this recombinant emerged in Melbourne in August 2015, but did not cause a significant increase of gastroenteritis until June 2016.

Another GII.4 recombinant, GII.P16/GII.4 Sydney 2012, emerged in mid-2015 and circulated in 2016, where it was then responsible for 12.5% of noroviruses identified in the Oceania region. This increase in strain activity was also observed in France between 2016 and 2017, where it was responsible for 24% of all norovirus outbreaks identified^41^. In addition, this strain became predominant in the United States, the United Kingdom^42^ and South Korea^43^ in 2017.

GII.17 was found to be the cause of increased gastroenteritis in Southeast Asian countries during winter of 2014^23,44,45^, but did not cause epidemics in other countries and only circulated at low prevalence. This is consistent with our findings where it accounted for less than 2.5% of norovirus cases in 2014/15 and only 4.6% of all noroviruses identified in Australia in 2016^25,26,46^. However, surprisingly, across all three WWTP sites in Sydney and Melbourne higher viral prevalence was observed with an average of 68.7% of all reads were attributed to GII.17 in the early months of 2016. These findings suggest GII.17 could be less virulent compared with other genotypes, which results in an underestimation of prevalence when only clinical samples are used for norovirus epidemiological studies.

A third recombinant virus, GII.P16/GII.2, circulated at a low prevalence (two cases in 2013^33^ and a single case in 2015) until June 2016, where it was responsible for 18.8% of all sporadic and outbreak cases identified in the Oceania region. This sudden increase of GII.P16/GII.2 was also observed in Germany, France and China. In Germany, it was found to be the most predominant strain between September and December 2016, accounting for 47.7% of acute gastroenteritis cases and 42% of outbreaks^47^. In France, GII.P16/GII.2 was responsible for 14% of outbreaks identified during the winter seasons of 2016 and 2017^41^. Similarly, this increase of GII.P16/GII.2 was observed in China, where it was first detected in August 2016 and caused 79% (44/56) outbreaks identified in November and December 2016^48^. Analysis of the VP1 full-length capsid sequences revealed no amino acid changes to account for the rapid predominance through antigenic variation. Therefore, amino acid substitutions in the GII.P16 polymerase have been proposed as a possible cause of the GII.P16/GII.2 emergence through changes in polymerase function^49^.

Norovirus GII.P21/GII.3 has been a major cause of childhood gastroenteritis since 2002^28,50^. In addition to the other emerging viruses detected, a novel GII.P12/GII.3 recombinant was identified in 2015 and 2016 in NZ and Australia, respectively. This recombinant was found to be associated with childhood infections and probably replaced the GII.P21/GII.3 recombinant childhood strain, as its prevalence declined rapidly from May 2015 onwards. The majority (90%) of Australian patients infected with GII.P12/GII.3 strain were under 12 years old, suggesting this virus has a predilection for infecting children. In 2016, this recombinant was detected at a prevalence of 8.1% in clinical samples compared with 0.57% of genotypes identified in waste water samples. We hypothesise that this is because children have lower norovirus immunity and infection would elicit more severe symptoms upon their first norovirus exposure. Therefore, children are more likely to visit a hospital or general practitioner for medical advice compared with adults.This is not without precedence, e.g., children with respiratory syncytial virus infection results in more severe outcomes and are present for medical intervention far more than adults^51^. Therefore, the use of clinical samples for surveillance could create bias towards viruses from children and selection viruses that induce symptoms, thus skewing genotypic distribution patterns.

Waste water is an ideal source for molecular epidemiological studies, because infected individuals excrete high levels of virus and continue to shed for up to 8 weeks, even after symptom resolution^52^. In the present study, NGS was used to examine the genetic diversity, prevalence and temporal dynamics of GII noroviruses present in complex waste water samples. The findings were compared with norovirus results obtained from clinical samples in Australia for 2016. A high level of norovirus genotypic diversity was identified in waste water samples from Sydney and Melbourne, with 18 of 22 capsid genotypes detected (GII.1-8, 10-14, 16, 17, 20-22), compared with 12 capsid genotypes detected in clinical samples. Among the four genotypes not identified, two were not expected (GII.18 and GII.19) as these only infect pigs^53^. The predominant GII genotype identified in waste water varied throughout the year; GII.17 was predominant in summer 2016 (January/February), which was slowly replaced by strains with a GII.4 capsid. From May 2016, GII.4 prevalence declined and the prevalence of GII.2 strains increased. This data correlated with an increase in GII.2-positive clinical samples, suggesting that waste water samples could augment clinical samples for norovirus surveillance. The increased abundance of GII.2 in wastewater was observed two months prior increased GII.2 detection in clinical samples, signifying its use as a predictive model, which could be used as a warning of impending epidemics leading to peaks of season.

To date, only a handful of studies have examined the genotypic diversity of viruses in wastewater, in which most used traditional Sanger sequencing/cloning methods^54-56^, with only two employing NGS technologies^57,58^. Studies by Kazama et al.^57^ and Prevost et al.^58^ applied second-generation sequencing technologies (pyrosequencing) to characterise norovirus diversity in waste water, which was then compared with the diversity found in clinical samples. A French study also identified a total of 16 norovirus GII genotypes of which GII.4 (86% Sydney 2012 variant) was the most prevalent in both sample types between May 2013 and May 2014^58^. In contrast to our study, Prevost et al.^58^ found a strikingly different distribution of norovirus diversity between waste water and clinical samples. For example, GII.4 was the predominant genotype identified in clinical samples throughout the year but only predominant in spring and winter in waste water samples.

In this study, we used 181 clinical samples collected over 2016 and matched the genotypes found to monthly genotype data from wastewater samples in three WWTPs. Kazama et al.^57^ also compared the norovirus diversity between waste water and clinical samples in a shorter timeframe, between November 2012 and March 2013. A relatively low number of clinical samples (*n* = 96) was used for correlation purposes, which only identified three GII genotypes (GII.4, GII.5 and GII.14) and a single GI virus (GI.6)^57^. In total, nine norovirus GII genotypes were identified in the waste water samples, with GII.14 as the most predominant (average of 36.2% of GII viruses identified), followed by GII.4 (21.5%). However, similar to our findings, GII.17 viruses were only detected in waste water samples and were not identified in clinical samples^57^. Further work is needed to determine the effectiveness of using waste water for norovirus molecular surveillance, but the initial studies show promising results.

In 2016, an unusual co-circulation of six prevalent norovirus strains was documented; this included four novel recombinant viruses, the GII.17 Kawasaki virus and the Sydney 2012 variant. Sequencing of both clinical and waste water samples demonstrated that both norovirus genotype diversity and the temporal dynamics of when certain viruses emerged coincided. The use of NGS technologies for monitoring norovirus diversity in waste water samples provided a more complete picture of norovirus epidemiology data population level to identify noroviruses associated more with asymptomatic infections, while complementing data obtained from clinical studies.
